# Resveratrol in Autism Spectrum Disorders: Behavioral and Molecular Effects

**DOI:** 10.3390/antiox9030188

**Published:** 2020-02-25

**Authors:** Michele Malaguarnera, Haroon Khan, Omar Cauli

**Affiliations:** 1 Research Center “The Great Senescence”, University of Catania, 95100 Catania, Italy; Michele.Malaguarnera@gmail.com; 2 Department of Nursing, University of Valencia, 46010 Valencia, Spain; 3 Department of Pharmacy, Abdul Wali Khan University, Marden 23200, Pakistan; haroonkhan@awkum.edu.pk; 4 Frailty and Cognitive Impairment Group (FROG), University of Valencia, 46010 Valencia, Spain

**Keywords:** antioxidant, animal model, natural compound, nutraceutical, developmental disorders

## Abstract

Resveratrol (RSV) is a polyphenolic stillbenoid with significant anti-oxidative and anti-inflammatory properties recently tested in animal models of several neurological diseases. Altered immune alteration and oxidative stress have also been found in patients with autism spectrum disorders (ASD), and these alterations could add to the pathophysiology associated with ASD. We reviewed the current evidence about the effects of RSV administration in animal models and in patients with ASD. RSV administration improves the core-symptoms (social impairment and stereotyped activity) in animal models and it also displays beneficial effects in other behavioral abnormalities such as hyperactivity, anxiety and cognitive function. The molecular mechanisms by which RSV restores or improves behavioral abnormalities in animal models encompass both normalization of central and peripheral immune alteration and oxidative stress markers and new molecular mechanisms such as expression of cortical gamma-amino butyric acid neurons, certain type of miRNAs that regulate spine growth. One randomized, placebo-controlled clinical trial (RCT) suggested that RSV add-on risperidone therapy improves comorbid hyperactivity/non-compliance, whereas no effects where seen in core symptoms of ASD No RCTs about the effect of RSV as monotherapy have been performed and the results from preclinical studies encourage its feasibility. Further clinical trials should also identify those ASD patients with immune alterations and/or with increased oxidative stress markers that would likely benefit from RSV administration.

## 1. Introduction

**Figure 1:**
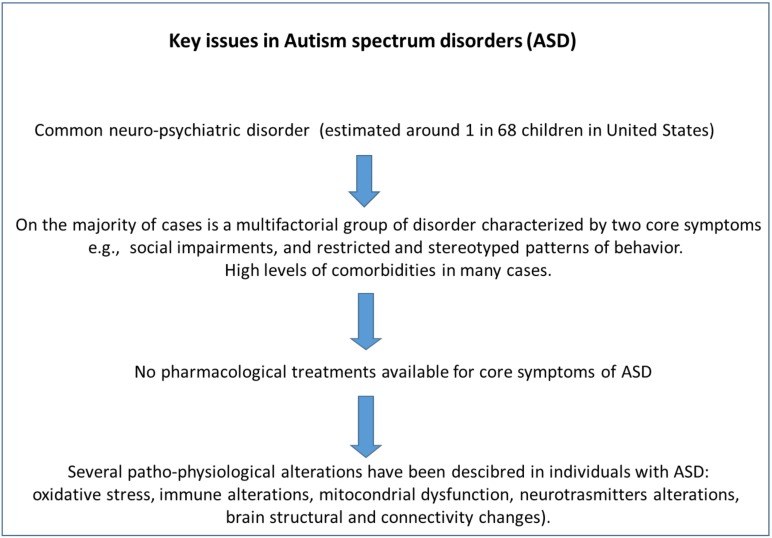
General scheme that summarizes the issues of autism spectrum disorder (ASD).

Autism spectrum disorder (ASD) has been defined as a set of developmental disabilities characterized by social impairments, communication difficulties and restricted and stereotyped patterns of behavior [1]. The prevalence of autism is estimated at around 1 in 68 children, with boys 4.5-fold more affected than girls in the United States [2]. Since the first description of clinical features of autism during the 1940’s [3], the molecular basis and the etiology have not been clear to the scientific community. Different research claims that there are a multitude of alterations associated with autism, but 25% of all cases of autism are related to these genes [4]. There are several causes that have been associated to the pathophysiology of ASD (Figure 1), among them the strongest evidence has been proven for immune dysregulation/inflammation and oxidative stress, followed by toxicant exposures and mitochondrial dysfunction measured in circulating blood leukocytes [5]. James et al. [6] found differences in the antioxidant capacity and in the concentration of several metabolites related with oxidative stress in 80 subjects with autism compared with healthy children.

Post-mortem brains from autistic subjects have shown altered levels of the transcriptome, some of them related to the inflammation and cytokine production [7]. The inflammation status of the brain is also reflected by an increase in pro-inflammatory cytokines in plasma [8] and the decrease of regulatory T cells [9] of ASD subjects. The impact of the regulatory cells (T-reg) in ASD is consonant with the investigation on the alteration of transcription factors expressed by T-reg cells in ASD spectrum. ASD is conditioned by a wide range of spectrum of behavior effect and despite that the exact molecular effects regarding the pathophysiology have not been identified with precision, oxidative stress and inflammation seem to play a role. For these reason, the natural compound resveratrol (RSV) due to its antioxidant and anti-inflammatory effects in several animal models of diseases has been proposed as a future treatment in ASD [10,11]. To date, the treatment of ASD is mainly based on behavioral therapy and there is not any pharmacological treatment for the core symptoms of ASD. In contrast, there are pharmacological treatments for the psychiatric comorbidities that frequently associate with ASD such as aggressive behaviors, epilepsy, sleep disorders and attention deficit hyperactivity disorder (among the most common comorbidities in ASD) (Table 1).

RSV (3,5,4-trihydroxy-trans-stillbene) is a polyphenolic stillbenoid which acts as a photoalexin and is produced naturally by several plants in response to attack by pathogens like bacteria and fungi [12]. It is commonly found in different fruits like berries and grapes, with direct and indirect antioxidant activity due to its interaction with other signal transduction pathways. In the last three decades, RSV has been extensively studied due to its antioxidant and anti-inflammatory properties [13]. The effects of this molecule have also been recently studied in several neurological diseases [14,15,16] because this molecule crosses the blood–brain barrier [17]. The aim of this review is to summarize the molecular mechanism and the effects of resveratrol on patients and animal models of ASD.

The main objectives of this review were:

(1) To evaluate the behavioral effects of RSV in animal models of ASD.
(2) To summarize the molecular mechanisms by which RSV administration improves behavioral deficits in animal models of ASD.
(3) To review clinical studies with RSV in patients with ASD and its adverse effects.

All original articles in the PubMed/Medline and Scopus electronic bibliographic databases up until December 30 2019 were analyzed for these purposes. The following inclusion criteria were applied: (1) full text in English, (2) primary articles only and (3) identification of data regarding resveratrol administration in animal models of autism or in patients with ASD. The title and abstract were analyzed to determine which articles to include. The full text was retrieved for those that fulfilled the inclusion criteria. Finally, the reference lists of all the relevant articles were manually cross-referenced to identify any additional articles. The primary search terms used were ‘resveratrol’ AND ‘autism*’.

## 2. Results

### 2.1. Behavioral Effects of RSV in Animal Models of ASD

Despite the fact that many genetic and environmental factors have been associated to ASD, the pathological mechanism remains unclear. The animal models mostly used to test the effect of potential drugs for ASD are pharmacological or used in genetically modified animals (Table 2). Regarding the pharmacological models, the most used in ASD preclinical research is the valproic acid (VPA) model [18]. VPA is a short-chained fatty acid widely used as an antiepileptic drug [19] and mood stabilizer [20,21]. This model came from epidemiological studies that associated a higher risk of ASD in mothers exposed to VPA during the pregnancy and the development has been associated to autism in humans [22]. For this reason, prenatal exposure to VPA in rats has been used as an approximation of ASD [23]. RSV administration (at doses of 3.6 mg/kg, subcutaneously for 12 to 13 days) prevented or reduced autistic-like social deficits in the VPA model [24,25,26] and sterotipic behaviors [27]. RSV at the same doses also prevented the reduced total reciprocal social interaction with a conspecific, the social transmission of food preference and repetitive self-grooming [26]. Propionic acid (PPA) is a short-chain fatty acid that is endogenous to the human body as both an intermediary of fatty acid metabolism and a fermentation end product of antibiotic-resistant enteric gut bacteria, such as clostridia. There are numerous lines of evidence suggesting that PPA and other enteric short-chain fatty acids may be elevated in ASD. Intracerebroventricular administration of PPA in rats have recently been used as an animal model of ASD [28]. Studies using this model have found that ICV treatment of PPA is capable of inducing numerous behavioral, electrophysiological and neuropathological changes in rats that are consistent with those observed in ASD. In addition, rats with intraventricular infusion of PPA have been demonstrated to be a good model reproducing characterizing molecular alterations found in ASD, such as neuroinflammation and oxidative stress [29]. In the PPA model, oral administration of RSV (5, 10, 15 mg/kg) normalized core symptoms of ASD e.g., the social interaction and stereotypy and also counteracted other behavioral alterations also reproduced in the PPA animal model, such as locomotor hyperactivity, anxiety, spatial learning, memory and depression-like behaviors [30]. The social transmission of food preference task (STFP) is based on the principle that dietary information can be communicated between rodents during social interaction [31]. Briefly, a demonstrator rodent (mice or rats) consumes a novel flavor, and then freely interacts with an observer rodent. The observer rodent is now “socially cued” toward that flavor and will prefer it in a choice paradigm over another novel “un-cued” flavor. Socially relevant cues are required for this transmission of food preference in rodents. VPA-treated rats presented a deficit in this test which is counteracted by RSV administration in rats (at doses of 3.6 mg/kg, administered subcutaneously for 12 days) [26]. Regarding the genetic model of ASD, RSV has been evaluated as a therapeutic agent in the BTBR model [32,33,34,35]. The BTBR is the only genetic model available so far in ASD preclinical research [36]. The BTBR mice model was originally bred for studies on insulin-resistance, diabetes-induced nephropathy and phenyloketonuria, and was identified only a decade ago as displaying strong and consistent autism-relevant behaviors [37,38,39]. The persistent self-grooming (as a surrogate of stereotyped and repetitive behavior observed in ASD patients) displayed by BTBR mice is reduced by RSV (administered intraperitoneally at doses of 20–40 mg/kg).

Among behavioral alterations found in ASD, an abnormal sensory processing is a key feature in ASD [40] and also prenatally VPA-exposed rats displayed such behavioral deficits. RSV administration (at doses of 3.6 mg/kg, subcutaneously) also prevented sensory deficits assessed by nest-seeking behavior and in a whisker nuisance task [25]. However, RSV has no effects on the delayed latency to reach the nest shavings and latency to make any choice (nest or sterile shavings) in VPA-treated animals [27]. More recently, several epidemiological studies showed the possible association between ASD and maternal hormone intervention [41,42]. As a result, prenatal and postnatal exposure with progestins were tested with RSV. RSV treatment at the dose of 20 mg/kg administered through oral gavage for 28 days restored the repetitive behavior tested through a marble burying paradigm in male rats, but not in females, where it was not impaired, and the social interaction in both genders [43].

### 2.2. Molecular Effects of RSV in Animal Models of ASD

The molecular mechanisms by which RSV prevented or reduced autistic-like behaviors in animal models are diverse and involve antioxidant effects, modulation of synthesis of anti-inflammatory molecules and new mechanisms relevant for ASD, such as those altering neuronal circuits and sensory-processing (Table 3). Regarding the antioxidant effects of RSV in an ASD model have been demonstrated in the PPA model [30]. PPA-treated rats displayed reduced brain glutathione content and enzymatic activities of superoxide dismutase and catalase Regular treatment with resveratrol (5, 10 and 15 mg/kg) produced a significant increase in the glutathione, superoxide dismutase and catalase levels in the brains of PPA-administered rats. Moreover, the daily administration of 15 mg/kg resveratrol produced a positive impact in increasing the reduced glutathione and catalase levels in comparison to 5 mg/kg and 10 mg/kg doses.

Regarding the immune-modulation and anti-inflammatory effects of RSV, several reports showed these effects in ASD models [30,32,33,34,35]. Bakheet et al. [33] studied the effect of RSV (20–40 mg/kg, intraperitoneally administered for 7 days). in the BTBR model by focusing on immune alteration and found that RSV beneficial effects were associated to several immune effects e.g., suppression of T helper 17 (Th17), T helper 2 and T helper 1 cell-related transcription factors and induction of Treg cell-related transcription factor. In another study of the same group, they found that BTBR mice showed higher levels of the chemokine receptors (CCR and CXCR), related to the inflammation, produced and expressed in CD4+ T cells than the B6 control mice did. Resveratrol treatment also decreased the mRNA expression levels of CCR and CXCR in the spleen and brain tissues and downregulated the chemokine receptor levels in CD4+ T cells [32]. This group also studied the impact of RSV treatment on Toll-like receptor pathways, which is increased in in CD4+ cells and brain of BTBR mice. They found that RSV decreased T lymphocytes CD4+, Toll-like receptors TLR2+, CD4+TLR3+, CD4+TLR4+, CD4+NF-κB+, and CD4+iNOS+ levels in spleen cells. RSV treatment decreased TLR2, TLR3, TLR4, NF-κB, iNOS and COX-2 mRNA and protein expression levels in brain tissue [34]. The inflammatory pathway related to the Janus kinase/signal transducers and activators of transcription (JAK/STAT) pathway and the expression of other pro-inflammatory cytokine like interleukin-6 (IL-6) and tumor necrosis factor alpha (TNF-alpha) in brain and in CD4+ cells, is overactivated in BTBR mice. The concentration of pro-inflammatory TNF-α is also increased in the PPA model [30]. Oral administration of RSV (5, 10, 15 mg/kg) antagonizes this molecular pathway and decreases the concentration of pro-inflammatory cytokine such as TNF-alpha, IL-6 and interferon IFN-gamma [30,35], thus counteracting some of the markers of neuroinflammation associated to ASD. Bhandari and Kuhad [30] analyzed several factors in the brain and justified the behavioral results through the beneficial effects of RSV, reducing oxidative stress markers like lipid hydroperoxyde and nitrite, and increasing the endogenous antioxidants: catalase, superoxide dismutase and glutathione. Neuroinflammatory response triggered by stimulation of matrix metalloproteinases (MMPs) also plays pivotal role in the development of autistic phenotype as MMPs stimulate inflammatory cytokines’ release along with mitochondrial deficits, which ultimately leads to neuronal dysfunction and precipitates autistic symptoms [44,45]. Supporting these observations found in ASD patients, oral RSV administration (5, 10, 15 mg/kg) improved the activity of brain mitochondrial complex enzymes and normalized levels of MMP-9 in the PPA model [30].

Bambini-Junior et al. [24] showed an unstable interaction between RSV and VPA by bioinformatics technique, indicating that the effects of RSV are due to its cellular mechanisms. The molecular mechanisms underlying the RSV (3.6 mg/kg, administered subcutaneously) prevention of sensory deficits in the VPA rat model may be through the possible recovery of the altered GABAergic neurons expressing parvalbumin (PVC-neurons) localization and cortical organization impaired in the primary somatosensory area and in the amygdala [25]. These brain areas play a major role on sensory processing of tactile stimulation of the whiskers in rodents [46].

MicroRNAs (miRNAs) are short, endogenous, noncoding RNAs that regulate gene expression through posttranscriptional mechanisms via degradation or inhibition of specific mRNAs targets [47]. Hirsch et al. [48] analyzed the miRNA concentration in the serum of a small group of children affected by ASD (from 5 to 15 years old) and VPA rats and the effects of RSV. They found that of miR134–5p and miR138–5p, both were increased in the serum of ASD patients. VPA rats also showed an augmentation of miR134–5p levels and RSV administration in VPA-treated rats prevented this effect. This miRNA inhibits the LIM domain kinase 1(Rac-LIMK1) pathway determining the reduction of actin polymerization and spine growth and the effects afforded by RSV might also be due to this effect. 

Progestins exposure during pregnancy cause reduced the estrogen receptor (ERβ) expression in the amygdala with autism-like behavior in the offspring. Postnatal or prenatal RSV oral treatment (20 mg/kg) significantly reversed this effect with ERβ activation. Xiu et al. [43] showed that RSV activates ERβ and its target genes by demethylation of DNA and histone on the ERβ promoter. It also decreased progestin-induced oxidative stress and ameliorated the impaired mitochondria and lipid metabolism in the amygdala neurons, subsequently improving ASD-like behavior.

### 2.3. Clinical Studies with RSV in Patients with ASD

Studies on ASD patients are still at the beginning stages. Hendouei et al. [49] recently published the results of a double-blind, placebo-controlled randomized trial (RCT) designed to assess the potential therapeutic effects of RSV plus risperidone on irritability in children (mean age 8 years old) with ASD. Both groups were treated with risperidone (a neuroleptic drug used in ASD to control associated-behavioral deficits) and the experimental group also received RSV 250 mg twice per day from the beginning of the study and the effects was compared to the placebo group that received only risperidone. All patients were assessed for ASD-related behavioral symptoms at baseline, week 5 and week 10 patients in the resveratrol group showed greater decline in hyperactivity/non-compliance score as a secondary outcome measure. In contrast, the improvements in primary outcome measure (irritability) and three secondary outcome measures (lethargy/social withdrawal, stereotypic behavior and inappropriate speech subscales) in the resveratrol group were statistically similar to those in the placebo group, which also improved.

The most frequent side effects observed in the RCT were restlessness (25.8%), constipation (19.3%) and diarrhea (19.3%) in the RSV group, and they were not significantly different form the prevalence of such adverse effects registered in the control group. Importantly, the safety of RSV concerned the frequency of extrapyramidal symptoms and weight gain induced by risperidone, which were similar between resveratrol and placebo groups.

## 3. Discussion

Apart from several behavioral and cognitive complications arising as a result of central nervous system dysfunction, there are various physiological comorbidities such as immune system deregulation, neuroinflammation, oxidative stress, mitochondrial dysfunction and gastrointestinal complications which can worsen existing behavioral complications. There are no available treatments for these physiological comorbidities. In the last years, several natural compounds like RSV, curcumin, or sulforaphane have been demonstrated to be effective against oxidative stress and immune function [50]. Specifically, RSV has been taken in consideration for brain diseases not just for its interesting biochemical features but also the ability to cross the blood–brain barrier. This compound has long been thought to be the explanation of the “French Paradox”, which consist in a lower incidence of cardiovascular disease despite a high saturated fat diet for its antioxidant properties and multidrug activities [51]. Oxidative stress is deeply involved in ASD pathology [52] because this dysregulation has been found and studied also as a possible biomarker in blood and urine [53,54,55]. There are reports that show the reduction in reduced glutathione, the major endogenous antioxidant, in several brain regions associated with communication, memory, sensory and motor coordination [45,56]. RSV acts as a scavenger against reactive oxygen species and reactive nitrogen species [57]. Moreover, RSV induces an increase of glutathione levels and acts on its metabolism through the activation of the nuclear factor erythroid 2-related factor 2 (NRF2) [58]. When it happens, NRF2 binds to the antioxidant response element (ARE), that increases the expression of different antioxidant enzymes. Glutathione enzymes result as impaired in the cell of the immune system of autistic children [59,60]. The beneficial effect of RSV against the oxidative stress shown in mice [30] may not just be caused from the scavenging activity and antioxidant induction through sirtuin (probably mainly SIRT1), a key regulator of metabolism and against oxidative stress. The activation of the pathway SIRT1/PGC-1α in the ASD lymphoblastoid cell lines determined a decrease in reactive oxygen species and an improvement of mitochondria, impaired in ASD [61]. Mitochondria damage in lymphoblastoid cell lines and the alteration in the fatty acid metabolism are co-factors related to ASD [62]. Peroxisome proliferation-activated receptor gamma (PPARγ), a ligand-activated transcription factor, has a wide spectrum of biological functions: regulating mitochondrial function, mitochondrial turnover, energy metabolism, antioxidant defense and redox balance, immune responses and fatty acid oxidation. Recently, it has been proposed as a therapeutic target to rescue mitochondrial function in neurological disease [63,64] and in ASD, where it was suggested to be modulated with RSV [11].

In order to understand the possible mechanism relative to ASD and the possible therapeutic treatment, it is necessary to analyze preclinical and clinical studies. Unfortunately, the majority of preclinical studies have engaged mainly male animals. Future research directed to the therapeutic effect of RSV are needed also in females. The reason for this choice is mainly epidemiological, in fact, it is well known that ASD affect males more than females by a ratio of 4:1. Hence, it suggests a potential role of sex hormones in the pathophysiology of this disorder. Alterations in the levels of estrogen receptors have been found in subjects with ASD [65,66,67]. This discovery led to the generation of a new animal model of ASD [42]. In fact, the prenatal exposure to progestins decreased ERβ expression in the brain with autism-like behavior, which was partially restored by RSV [43]. RSV interacts with estrogen receptors and act as an ERα ligand, which aids to modulate the inflammatory response but not cell proliferation [68].

The immune system is another important cofactor of ASD. Several reports show that the immunophenotype of ASD patients is altered [69] with an augmented amount of Th17 cells [70]. Different transcription factors related to the immune system have been recently associated to ASD, in fact, it was shown that the maternal Th17 cells, through the interleukin-17a pathway, promote autism-like phenotypes in offspring in mice [71]. Hu et al. [72] reported higher levels of GATA binding protein 3 (GATA-3), a transcription factor, in lymphoblastic cell lines derived from the lymphocytes of autistic children, with respect to that of their non-autistic siblings. In another manuscript, the Toll-like receptor (TLR) pathway is activated during inflammation and neuroinflammation in ASD [73]. These studies highlight the importance of the immune system during the development, its implication on the ASD symptoms and suggest the beneficial implication of a treatment with RSV.

Despite the beneficial effects, RSV during pregnancy may cause abnormalities in the fetus, as shown in Japanese macaques [74]. According to this study, it is necessary to adjust its concentration in order to understand its applicability in clinical trials.

The therapeutic effects of resveratrol on the central nervous system (CNS) have been investigated by a number of clinical trials in neurodegenerative disorders, including mild cognitive impairment (MCI) (NCT01219244), Alzheimer’s disease (AD) (NCT01504854), Parkinson’s disease (PD) (NCT03091543) and Huntington disease (HD) (NCT02336633). The effects of RSV as a monotherapy in ASD patients has not been studied so far. In contrast, its effects on irritability has been evaluated in an RCT performed on children with ASD also receiving the anti-psychotic drug risperidone [49]. Irritability is probably the most damaging associated symptom of ASD, which might complicate treatment of patients both at home and in clinical settings [75,76]. In this RCT, RSV failed to enhance the effects afforded by risperidone and no effects on core symptoms of ASD e.g., social communication deficits and stereotype/limited activities, were observed. ASD is considered as a heterogeneous disorder with considerable variability among ASD subjects. Of note, diversity among ASD patients might lead to different clinical responses, even when the same therapies and interventions are used. Although resveratrol could not improve the majority of ASD-associated behavioral impairments, its alleviation of hyperactivity/non-compliance is an important issue and the resulting molecular mechanisms might be due to its action on several pathological underpinnings of ASD. Comorbid attention deficit hyperactivity disorder (ADHD) with ASD is very common (a prevalence ranging between 26–70%) [77,78,79,80]. The beneficial effects of resveratrol administration co-administered with risperidone on hyperactivity/noncompliance deficits may be beneficial for a subgroup of ASD patients with comorbid ADHD or its subtype (hyperactive/impulsive, inattentive or combinations) and further RCT should focus on this subgroup of ASD patients besides evaluating the drug as a monotherapy.

## Figures and Tables

**Table 1:** Pharmacological treatment for psychiatric comorbidities in autism spectrum disorder (ASD) patients.

Comorbidity	Drug Class
Hyperactivity/inattention	Psychostimulants
	Non-stimulants
Sleep alterations	Hormone (Melatonin)
	Antihistamines
Irritability	Atypical antipsychotics
Epilepsy	Antiepileptics
Aggression	Atypical antipsychotics
Miscellaneous	Antidepressants (selective serotonin reuptake inhibitors)
	Mood stabilizers

**Table 2:** Behavioral effects of resveratrol (RSV) in animal models of ASD.

Study	Animal Model	Dose RSV, Treatment Duration and Route of Administration	Behavioral Alterations	Main Outcome
Bambini-Junior et al., 2014 [24]	Prenatal exposure of valproic acid in Wistar rats	3.6 mg/kg, subcutaneous. Administered daily for 13 days.	Social memory and preferences (Three chamber sociability and social novelty test).	RSV prevents autistic-like social behaviors.
Bakheet et al., 2016 [32]	BTBR model	20–40 mg/kg, intraperitoneally For 7 days.	Self-Grooming (repetitive behavior).	RSV reduced repetitive behavior.
Bhandari and Kuhad 2017 [30]	Propanoic acid (PPA) infused into the anterior portion of the lateral ventricle in Sprague-Dawley rats	5, 10, 15 mg/kg. Oral treatment. Administered daily for 27 days after PPA infusion.	Social interaction, stereotypy, locomotor activity, anxiety, spatial learning, memory, depression-like behaviors.	RSV normalizes the social interaction, stereotypy, locomotor activity, anxiety, spatial learning, memory and depression-like behaviors.
Fontes-Dutra et al., 2018 [25,27]	Prenatal exposure of valproic acid in Wistar rats	3.6 mg/kg, subcutaneous. Administered daily for 12 days.	Effect on sensory behavior (Nest-seeking behavior and in whisker nuisance task).	RSV improves the percentage of correct choices (reach the nest shavings) per litter.
Hirsch et al., 2018 [26]	Prenatal exposure to valproic acid in Wistar rats	3.6 mg/kg, subcutaneous. Administered daily for 12 days.	Reciprocal social interaction test (Social Transmission of Food Preference test).	RSV counteracts the deficit in the social interaction test.
Xie et al. 2018 [43]	Prenatal and Postnatal exposure to different progestins, and then just norethindrone (20 mg) in Sprague-Dawley rats	20 mg/kg administered through oral gavage for 28 days (two protocol: prenatal and postnatal treatment).	Repetitive behavior, anxiety, and social interaction test.	RSV recovers the repetitive behavior (Marble burying test) and the deficit found in social interaction test.

**Table 3:** Molecular effects of RSV in animal models of ASD.

Study	Animal Model of ASD	Dose RSV, Treatment Duration and Route of Administration	Molecular Effects of RSV in ASD Models
Bakheet et al., 2016 [32]	BTBR model	20–40 mg/kg, intraperitoneally administered for 7 days.	Decreases the expression (mRNA) levels of CCR and CXCR in the spleen and brain tissues and downregulated the chemokine receptor levels in CD4+ T cells.
Bakheet et al., 2017 [33]	BTBR model	20–40 mg/kg, intraperitoneally administered for 7 days.	Suppression of upregulation of T helper 17 (Th17), T helper 2, and T helper 1 cell-related transcription factors and induction of T-reg cell-related transcription factor such as FOX-p3, GATA.
Bhandari and Kuhad 2017 [30]	Propanoic acid (PPA) infused into the anterior portion of the lateral ventricle in Sprague-Dawley rats	5, 10, 15 mg/kg. Oral treatment. Administered daily for 27 days after PPA infusion.	Increase the concentration of reduced glutathione, superoxide dismutase and catalase in the brain. Reduction of oxidative stress markers (lipid hydroperoxyde and nitrites). Normalizes brain levels MMP-9 and TNF-alpha.
Ahmad et al., 2018 [34]	BTBR model	20–40 mg/kg, intraperitoneally administered for 7 days.	Decreases TLR2, TLR3, TLR4, NF-κB, iNOS, and COX-2 mRNA and protein expression levels in brain.
Ahmad et al., 2018 [35]	BTBR model	20–40 mg/kg, intraperitoneally administered for 7 days.	Decreases IL-6, TNF-alpha, IFN-gamma and STAT-3 expression in spleen and in the brain.
Fontes-Dutra et al., 2018 [25,27]	Prenatal exposure of valproic acid in Wistar rats	3.6 mg/kg, subcutaneous. Administered daily for 12 days.	Restoration of GABAergic neurons and cortical organization in the primary somato-sensory area and in the amygdala.
Hirsch et al., 2018 [26]	Prenatal exposure of valproic acid in Wistar rats	3.6 mg/kg, subcutaneous. Administered daily for 12 days.	Prevention of the augmentation of miR134–5p levels induced by valproic acid.
Xie et al. 2018 [43]	Prenatal and postnatal exposure to different progestins in rats	20 mg/kg of through oral gavage for 28 days (two protocol: prenatal and postnatal treatment)	Augmentation of estrogen receptor (ERβ) expression and its target genes by demethylation of DNA and histone on the ERβ promoter.

The CCR chemokine receptors are expressed on cells important to allergic inflammation including eosinophils, basophils, lymphocytes, macrophages, and dendritic cells, whereas the CXCR are expressed mainly on neutrophils and lymphocytes. FOXP3 (forkhead box P3), also known as scurfin, is a protein involved in immune system responses. A member of the FOX protein family, FOXP3 appears to function as a master regulator of the regulatory pathway in the development and function of regulatory T cells. CCR, CXCR: chemokine receptors (or beta chemokine receptors); GATA, STAT-3: transcription factors; COX: Cyclooxygenase; iNOS: Inducible nitric oxide synthase; PPA: propionic acid; GABA: Gamma-amino butyric acid; TLR: Toll-like receptors.
